# Applications of Artificial Intelligence in Emergency Departments to Improve Wait Times: Protocol for an Integrative Living Review

**DOI:** 10.2196/52612

**Published:** 2024-04-12

**Authors:** Bahareh Ahmadzadeh, Christopher Patey, Oliver Hurley, John Knight, Paul Norman, Alison Farrell, Stephen Czarnuch, Shabnam Asghari

**Affiliations:** 1 Centre for Rural Health Studies Faculty of Medicine Memorial University of Newfoundland St. John's, NL Canada; 2 Eastern Health Carbonear Institute for Rural Reach and Innovation by the Sea Carbonear General Hospital Carbonear, NL Canada; 3 Faculty of Medicine Memorial University of Newfoundland St. John’s, NL Canada; 4 Data and Information Services Digital Health NL Health Services St. John’s, NL Canada; 5 Division of Community Health and Humanities Faculty of Medicine Memorial University of Newfoundland St. John’s, NL Canada; 6 Health Sciences Library Memorial University of Newfoundland St. John’s, NL Canada; 7 Department of Electrical and Computer Engineering Faculty of Engineering and Applied Science Memorial University of Newfoundland St. John’s, NL Canada; 8 Discipline of Emergency Medicine Faculty of Medicine Memorial University of Newfoundland St. John’s, NL Canada

**Keywords:** emergency department, ED, wait time, artificial intelligence, AI, living systematic review, LSR

## Abstract

**Background:**

Long wait times in the emergency department (ED) are a major issue for health care systems all over the world. The application of artificial intelligence (AI) is a novel strategy to reduce ED wait times when compared to the interventions included in previous research endeavors. To date, comprehensive systematic reviews that include studies involving AI applications in the context of EDs have covered a wide range of AI implementation issues. However, the lack of an iterative update strategy limits the use of these reviews. Since the subject of AI development is cutting edge and is continuously changing, reviews in this area must be frequently updated to remain relevant.

**Objective:**

This study aims to provide a summary of the evidence that is currently available regarding how AI can affect ED wait times; discuss the applications of AI in improving wait times; and periodically assess the depth, breadth, and quality of the evidence supporting the application of AI in reducing ED wait times.

**Methods:**

We plan to conduct a living systematic review (LSR). Our strategy involves conducting continuous monitoring of evidence, with biannual search updates and annual review updates. Upon completing the initial round of the review, we will refine the search strategy and establish clear schedules for updating the LSR. An interpretive synthesis using Whittemore and Knafl’s framework will be performed to compile and summarize the findings. The review will be carried out using an integrated knowledge translation strategy, and knowledge users will be involved at all stages of the review to guarantee applicability, usability, and clarity of purpose.

**Results:**

The literature search was completed by September 22, 2023, and identified 17,569 articles. The title and abstract screening were completed by December 9, 2023. In total, 70 papers were eligible. The full-text screening is in progress.

**Conclusions:**

The review will summarize AI applications that improve ED wait time. The LSR enables researchers to maintain high methodological rigor while enhancing the timeliness, applicability, and value of the review.

**International Registered Report Identifier (IRRID):**

DERR1-10.2196/52612

## Introduction

### Background

Extended emergency department (ED) wait times are a major health care system problem worldwide [1-4]. Long wait times in the ED can threaten patients’ well-being, leading them to depart the ED without receiving the essential care they require. Additionally, this situation contributes to overcrowding within the ED and fosters a sense of dissatisfaction among both patients and ED personnel [2]. Previous studies have investigated many initiatives to reduce ED wait times. Among them, a new approach is the use of artificial intelligence (AI) [3,4].

AI is one of the most important technological advancements of the Fourth Industrial Revolution [5]. AI refers to the use of technology and computers to mimic human-like critical thinking and intelligent behavior. In 1956, the word AI was first used by John McCarthy to refer to the science and engineering of creating intelligent machines AI refers to the use of technology and computers to mimic human-like critical thinking and intelligent behavior. As mentioned by Amisha et al [6], John McCarthy used the word AI for the first time in 1956 to refer to the science and engineering of creating intelligent machines. Recent years have seen exponential growth in the technological and scientific aspects of AI as well as machine learning, one of its main subcategories [7]. Notable benefits include increased productivity and innovation. Significant progress has been made to date in several fields, including computer vision, natural language processing, audio analysis, smart sensing, and many more [8]. Therefore, modeling based on AI is the key to creating automated, intelligent, and smart systems that meet today’s needs. Different forms of AI, including analytical, functional, interactive, textual, and visual AI, can be used to improve an application’s intelligence and capabilities to solve problems in the real world [5]. Although many of AI’s practical applications are still in the early stages and require further research and development, the technology has the potential to revolutionize medicine in ways that have not yet been considered [6].

Harnessing the power of AI holds promising potential to improve the quality of care within EDs by effectively tackling challenges such as overcrowding by offering advanced clinical decision-making tools [9,10]. By developing an AI-assisted module, a significant reduction in wait times for outpatient services was demonstrated in the ED, according to a retrospective cohort study [4]. Furthermore, a prospective study revealed a notable decrease in the wait time for receiving care services through digital automation [11].

All AI methods that enable computers to learn from data without explicit programming are included in machine learning [12]. An Italian study used 2 large data sets of EDs to test several machine learning methods using predictive analytics. The findings demonstrate the viability of a real-time performance monitoring system that supports operational decision-making and has major practical implications for EDs and hospitals [13]. An Australian study showed that wait time forecasts for low-acuity ED patients assigned to the waiting room were improved through machine learning techniques and a wide range of queuing and service flow features. Machine learning models surpass the best rolling average in terms of mean absolute prediction error using queuing and service flow characteristics along with knowledge of daily fluctuations, and quantile regression lowers the proportion of patients with significantly underestimated wait times [14].

Deep learning is a subclass of machine learning defined primarily by neural network models with more layers and, in general, more neurons than typical machine learning neural networks. Additionally, deep networks, relative to traditional neural networks, achieve increased performance with increasingly large amounts of data, becoming practically realizable due to modern advances in computing power [15]. In a study conducted in Saudi Arabia, deep learning was used to forecast the length of time patients would wait in the ED’s queue system. The findings of this study demonstrated the applicability of deep learning models for predicting patient wait times in the ED [16].

By looking at current developments in ED operations and clinical patient management, the authors of a review paper summarized the applications of AI in emergency medicine. They came to the view that the areas of prehospital emergency management, patient acuity, triage, and disposition, prediction of medical diseases and conditions, and ED management are where AI applications in ED are most prevalent [2]. Another systematic review study sought to show how AI was applied in ED and how it changed how ED practitioners’ work was organized. Most AI applications, according to the study’s findings, involved AI-based tools to support clinical judgment and reduce the pressure on overburdened EDs. Additionally, AI support was primarily provided during triage, the decision-making stage that determines a patient’s course, and there is strong evidence that AI-based apps could enhance clinical decision-making [10].

Based on our understanding of the literature, the systematic reviews that encompassed studies involving AI applications in the context of EDs were comprehensive and addressed diverse aspects of AI implementation. However, these reviews were limited in their usefulness due to the absence of a plan for regular updates on AI progress, rendering them less applicable across some contexts. It is crucial to recognize that AI development is a revolutionary field with a rapidly evolving landscape. As such, reviews in this domain must undergo frequent updates to stay relevant. As a result of AI, computer programs can answer questions intelligently and infer facts based on real-world data. AI will become a core component of all contemporary decision-making in the immediate future. To keep up with the ongoing changes and revolutions in this field, we plan to conduct living systematic reviews (LSRs). Our review will be regularly updated. We will incorporate relevant new evidence when it is available.

### Study Goals and Objective

The study aims to summarize the available evidence on how AI can impact ED wait times; describe the applications of AI in reducing wait times; and examine the depth, breadth, and quality of evidence related to the application of AI in reducing wait time in the ED.

## Methods

### Rationale for LSR

LSR enables researchers to maintain high methodological rigor while enhancing the timeliness, applicability, and value of the review. Our strategy involves conducting continuous monitoring of evidence, with biannual search updates and annual review updates. Upon completing the initial round of the review, we will refine the search strategy and establish clear schedules for updating the LSR.

### Type of Review

This will be an integrative review. Integrative reviews stand out as the most exhaustive form of systematic review methodology. They offer the flexibility of diverse sampling strategies and a broad scope of objectives, enabling them to provide a comprehensive representation of complex ideas, theories, or significant health care issues [17]. We selected this type of review since we aimed to examine the full breadth of techniques, methods, algorithms, and modalities of AI used to improve wait time in EDs from various academic sources. As mentioned by Hopia et al [18], according to Whittemore and Knafl’s methodological steps, the integrated review method can use diverse data sources, thereby creating a comprehensive understanding of the topic of interest by presenting the state of the science and contributing to theory development. Whittemore and Knafl [17] approach consists of 5 stages and is based on Cooper’s theoretical framework, which is one of the methodological approaches used in integrative reviews. These steps generally include problem identification, literature search, data evaluation, data analysis, and presentation [17].

### Systematic Review Team

Research team members participating in the systematic review include researchers, physicians, and nurses with expertise in emergency medicine, librarians, learners, patients, AI specialists, and review methodologists.

### Patient Engagement

Our research plan and related materials will be showcased to SurgeCon’s [19] patient engagement working group, comprising individuals from diverse backgrounds, including people of different gender identities residing in urban and rural areas of Newfoundland and Labrador. This approach aims to comprehensively gather the needs, desires, and firsthand experiences of those who stand to gain from the implementation of AI in improving ED wait times.

### Protocol of the Integrative Review

#### Overview

**Figure 1:**
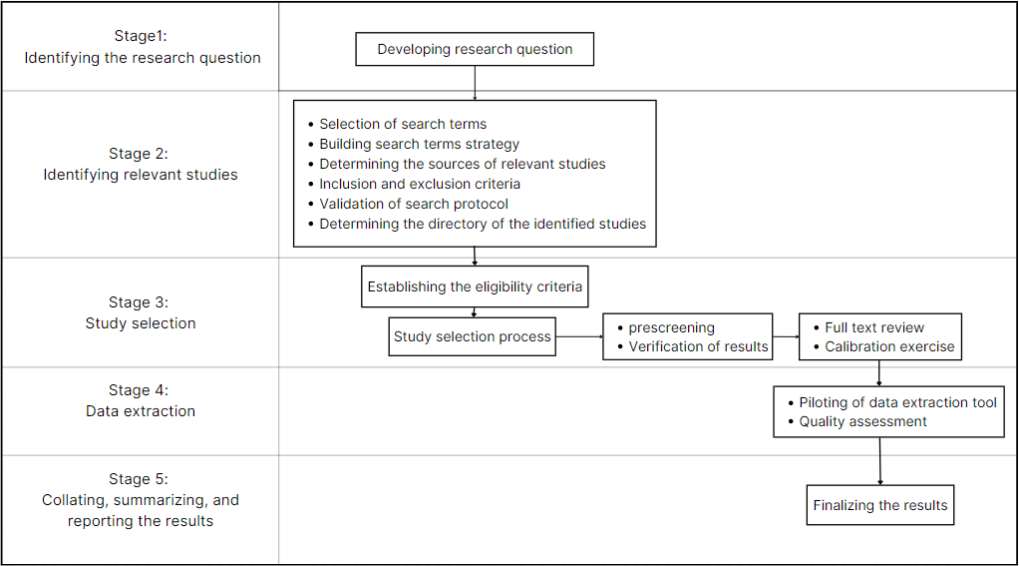
Visual representation of the 5 key stages in an integrative review protocol.

Our designed integrative review protocol will include 5 stages summarized in Figure 1, which will be explained in the following. Furthermore, to ensure transparent and accurate reporting, we will use PRISMA (Preferred Reporting Items for Systematic Reviews and Meta-Analyses) guidelines in this review [20].

#### Stage 1: Identifying the Research Question

Our research questions will be developed and refined by a team of researchers and clinicians, including physicians and nurses with expertise in emergency medicine, patients, and librarians. “How does the application of AI reduce ED wait times, as supported by evidence from current literature?”

#### Stage 2: Identifying Relevant Studies

##### Selection of Search Terms

The search terms will be developed by content experts and patients. These terms include general variations on “ED,” “wait times,” “AI,” “machine learning,” “deep learning,” and any more specific terms related to algorithms and methods used in the field of AI and indicators related to wait times in the ED. Once completed, the research question’s key terms will be chosen by relevant stakeholders, which will be compiled alongside a list of potential synonyms or other terms identified by a librarian. The optimal search phrases will be determined by searching for Medical Subject Heading (MeSH) terms, the MeSH tree, and related words in keywords and references.

##### Building Search Terms Strategy

A librarian will assist in exploring various word combinations in databases to discover the most effective search method. When searching relevant literature, search phrases are iteratively improved by evaluating various terms, merging new terms, and finding new relevant citations. The MeSH and keywords will be combined in the search. For other data sets, different search techniques will be applied as needed.

##### Sources of Relevant Studies

We initiate the review process by conducting a comprehensive examination of peer-reviewed papers cited in electronic databases. This meticulous approach allows us to identify and encompass all relevant, available information sources. A librarian will provide support in completing the search for research studies in the following databases for studies that were published between January 1, 1946, and August 17, 2023: Embase, MEDLINE via Ovid, CINAHL, Cochrane, and Scopus. The search strategy for MEDLINE is available in Multimedia Appendix 1.

##### Inclusion and Exclusion Criteria

During our search for relevant research papers, we will incorporate peer-reviewed papers and published PhD dissertations from reputable repositories. Studies that focus on AI-related concepts, including AI algorithms and techniques, in the context of EDs to improve wait times will undergo a full-text review. We have categorized all the AI-related concepts and terminology we want to explore in reducing ED wait times in Multimedia Appendix 2. Since AI and its applications are evolving, we will reevaluate the inclusion and exclusion criteria in the next review to ensure new terminologies for AI will be included in our research. Our inclusion criteria will consist of studies that specifically concentrate on the application of AI techniques in the ED to decrease wait times. These studies must include wait times as an outcome measure and present relevant results. We will exclude studies that do not directly address the use of AI or do not primarily focus on reducing wait times in the ED. Additionally, master theses and conference abstracts will also be excluded from our selection.

##### Validation of the Search Protocol

The gold standard papers and journals recommended by subject matter experts will be used to test the search methodology and calibrate our search technique. The eligibility requirements will be changed as necessary. The search strategy for this LSR will be iteratively revised, and new search terms may supplement future searches if required.

##### Directory of Identified Studies

Covidence software (Veritas Health Innovation) will be used to manage the review and build a directory of publications.

#### Stage 3: Study Selection

##### Overview

The process of inclusion studies will be iterative and involve searching the literature, adjusting the search strategy, determining eligibility, prescreening, reviewing the full text of the literature for inclusion, and keeping only studies that discuss the use of AI to reduce ED wait times.

##### Eligibility Criteria

Members of our team who are knowledgeable about AI and ED and who will be blinded to the study results in question will make decisions about the review process methodology. Inclusion criteria will ensure a wide range of literature from various sources.

We will include patients, regardless of gender or age, who have been analyzed by AI algorithms. Audits or anecdotal information, planning-stage research, pilot studies, undergraduate dissertations, book reviews, gray literature, such as unpublished theses and reports from relevant websites, and policy assessments are among the exclusion criteria. We will look at both qualitative and quantitative studies. If the paper is a systematic review, all included studies in the review will be examined, and the related ones will be included in our review. In addition, we will conduct backward and forward citations of all studies eligible for data extraction in our review.

##### Study Selection Process

###### Prescreening

Papers will be digitally stored and managed in Covidence software, and duplicates will be removed by Covidence software. After training team members, they will independently review the titles and abstracts of all publications found through database searches to determine eligibility. Conflicts will be resolved through a discussion between the 2 reviewers (BA and AG); a third team (SA) member will provide feedback when necessary. If studies or abstracts do not address the topic of the search, or if the studies are commentaries or editorials, they will be deemed unrelated and excluded.

##### Verification of Results

One of the 2 reviewers (BA and AG) will then reevaluate a random sample of 5% (n=875) of the papers that were rejected based on title and abstract to make sure that all pertinent studies were considered. All excluded papers will be reexamined if more than 5% (n=44) of the sample is determined to be relevant. We will also provide reviewers with retraining as needed.

##### Full-Text Review

Two research team members (BA and AG) will independently review all full texts. Only English papers are subject to full-text reviews, although the total number of studies considered appropriate by title, abstract, and full text will be noted for future use.

##### Calibration Exercise

We will conduct a calibration exercise before the process begins and then continue the calibration exercise throughout the review. Five percent of the listed citations will be randomly chosen. Two reviewers (BA and AG) will independently assess the full text against the eligibility criteria provided in Covidence. They will then discuss their rationale for including or excluding each article, and a third team member (SA) will provide feedback if necessary. Using an iterative process, if the level of agreement between the 2 reviewers is low (0.5), the eligibility criteria for screening and full-text review and the exclusion criteria for full-text review will be revised on Covidence. Then, the remaining citations will be evaluated by the reviewers (BA and AG), and a third reviewer (SA) will settle any disagreements. Furthermore, we will schedule biweekly team meetings to assess the review process. We will examine the reviewers’ understanding of the eligibility criteria during these meetings and provide training if needed. The reviewers need to maintain a 0.8 agreement during the process.

#### Stage 4: Data Extraction

##### Overview

A data extraction tool for systematic data gathering from the indicated studies will be prepared in Excel (Microsoft Corporation). The tool will be made to extract data based on citation type (eg, original research), country, study date, study methodological features, study design, study population, sample size, AI techniques used, input variables, wait time metrics, outcomes measured, results performance, performance measure, and any limitations or challenges reported by the authors.

##### Piloting of the Data Extraction Tool

The team evaluates the data extraction tool by using a random sample of 5% (2) of the included studies. If required, the data extraction tool will also undergo frequent revisions. The data will be reviewed and extracted separately by 2 independent reviewers (BA and AG).

##### Quality Assessment

A scoring system for systematic reviews of mixed research will be used to evaluate the quality of primary studies (both quantitative and qualitative) [21]. The goal of the quality assessment is to determine the overall caliber of the studies in the sample rather than to identify or reject weaker studies. The quality of the screened studies will be reported.

#### Stage 5: Collating, Summarizing, and Reporting the Results

We will use a complex strategy to understand and combine the results of many studies for an interpretive synthesis. A descriptive summary of the identified and relevant studies will be reported. The frequency of research with study designs that match the criteria included in the data extraction tool will be provided [22]. In addition to collecting results, we seek to uncover underlying meaning, patterns, and relationships in the data. The framework developed by Whittemore and Knafl [17] is a useful resource for accomplishing this interpretative synthesis. Detailing the documented outcomes of AI interventions and the kinds of AI methods used by our data extraction tool’s specifications will be part of the interpretive synthesis. We aim to identify commonalities or patterns in the studies by categorizing their results according to their impact on various wait time metrics. We will classify the findings of the identified research according to how they affect different wait time metrics in the ED using interpretive synthesis. These metrics include length of stay, number of patients leaving without being seen by a physician or their delegate, time to the initial physician assessment, and others. We will list the reported effects of AI interventions and the types of AI techniques implemented based on the criteria included in our data extraction tool. For an in-depth visual representation, the outcomes of the interpretative synthesis will be presented using descriptive tables, frequency tables, and diagrams. To develop a combined estimate of the effectiveness of AI initiatives in lowering ED wait times, the integrative review will evaluate the included papers’ suitability for conducting a meta-analysis. If the selected studies show sufficient homogeneity in their methods and findings, a meta-analysis could be possible and provide a quantitative synthesis of the findings. We improve the breadth and clarity of this study’s results by integrating these components into our interpretive synthesis approach, offering a robust analysis of the effect of AI interventions on ED wait times.

### Knowledge-User Consultation

The review will be carried out using an integrated knowledge translation strategy, and knowledge users will be involved at all stages of the review to guarantee applicability, usability, and clarity of purpose. This strategy aims to engage content experts and the community advisory committee within our team through multiple consultations. The objective is to actively involve them in shaping the study’s outcomes, action plan, and research agenda while also fostering opportunities for knowledge exchange [22,23]. We will organize regular meetings with the knowledge users’ group to provide an opportunity to receive their feedback throughout the review. In addition, we will regularly update the progress of the review to notify them. By adopting these strategies, we can ensure that feedback from consultations is systematically integrated into the integrative review process, enhancing the relevance and applicability of the study outcomes. This team includes researchers and clinicians, including physicians and nurses with expertise in emergency medicine, learners, patients, AI specialists, and review methodologists. A practical understanding of the difficulties and possible advantages of applying AI to reduce wait times can be gained by speaking with ED physicians, nurses, administrators, and other health care workers who have firsthand experience working in the ED. Speaking with those in charge of planning the ED system and allocating resources can give us an understanding of the broader effects of using AI in the ED. They can provide viewpoints on financing priorities, legislative considerations, and the viability of incorporating AI solutions into current health care systems. Speaking with professionals involved in the development and application of AI in similar contexts will help us comprehend the technical facets and capabilities of AI systems on a deeper level. These professionals can offer perceptions of the promise of AI technologies, the difficulties in putting them into practice, and factors to consider for successful integration into the ED environment. This LSR aims to provide updated information on the application of AI to improve ED wait times. Initial review findings will be regularly communicated to our team to validate our conclusions and help direct the completion of the review [22]. In addition to scientific papers and academic presentations, a summary of potential clinical practice implications will be created using the study’s findings, including any areas that might call for medium- or long-term action. The summary will be distributed in our biweekly meetings with ED stakeholders to generate ideas, formulate research questions, and decide on suitable approaches for the SurgeCon ED wait time improvement team in Newfoundland and Labrador, Canada.

## Results

The literature search was completed by September 22, 2023. We identified 17,569 studies. The title and abstract screening was started independently by 2 reviewers (BA and AG) on September 23, 2023, and ended on December 9, 2023. The full-text review phase started with 70 eligible papers on January 22, 2024, and is in progress.

## Discussion

Recognizing the dynamic nature of AI research and its impact on health care decision-making in emergency medicine, we propose the use of LSRs. The major goal of LSRs is to keep our reviews up-to-date and relevant by including new information and revolutions in the field of AI as they become available. Using LSRs, we propose a methodological innovation that maintains rigor and assures that reviews remain relevant and applicable over time, reflecting the continual progress of AI applications in EDs.

Our research aimed to analyze the published studies examining AI applications in EDs, with a particular emphasis on reducing wait times. The analysis of the literature found that studies of AI applications in EDs to improve wait times cover a wide range of topics in emergency medicine [4,9,11,13,14,16]. We also found a few systematic reviews showing that scholars and specialists in the field have been proactive in synthesizing the available knowledge [2,10]. Despite their comprehensiveness, these systematic assessments have 1 significant limitation: no plan for regular updates. Since the field of AI is characterized by rapid change and continual progress, this issue jeopardizes the use of these studies in future settings. AI technologies are continually improving, and their applications in health care, including EDs, are no exception.

The study has several limitations. Implementing an LSR strategy may necessitate changes to existing review procedures as well as potential challenges in gathering and synthesizing constantly emerging data. Furthermore, the feasibility and practicality of conducting living reviews must be carefully considered. In addition, like other types of systematic reviews, the LSR is prone to publication bias, and the quality of the synthesis could be affected by the quality of the included studies.

In conclusion, our study illuminates the current state of AI applications in EDs and addresses a crucial gap in systematic review methods. LSRs emerge as a timely solution to the challenge of obsolescence amid rapid AI growth and advancements. Embracing LSRs ensures timely information for health care decision makers and sets a precedent for enhancing review techniques in the dynamic realm of AI research. As major technological shifts loom, our study highlights the methodological evolution needed to fully harness AI’s potential in health care.

## Appendix

Multimedia Appendix 1 Search strategy.

Multimedia Appendix 2 Related concepts and terms.

Multimedia Appendix 3 The original ChatGPT transcript.

## Abbreviations

AI: artificial intelligence
ED: emergency department
LSR: living systematic review
MeSH: Medical Subject Heading
PRISMA: Preferred Reporting Items for Systematic Reviews and Meta-Analyses

## Data Availability

The data sets generated and analyzed during this study are available from the corresponding author upon reasonable request.
