# Supplementary material for: Applications of Artificial Intelligence in Emergency Departments to Improve Wait Times: Protocol for an Integrative Living Review
Source: JMIR Res Protoc. 2024 Apr 12;13:e52612. doi: 10.2196/52612 (PMC11053385; doi:10.2196/52612)
Supplement: Multimedia Appendix 1 [file resprot_v13i1e52612_app1.pdf]

Ovid MEDLINE(R) and Epub Ahead of Print, In-Process, In-Data-Review & Other Non-Indexed Citations and Daily <1946 to August 17, 2023>

1 exp Artificial Intelligence/ 177295

2 (artificial intelligence or deep learning or machine learning or fuzzy logic or machine intelligence).tw,kf. 156136

3 exp Emergency Medical Services/ 169557

4 (ER or ED).tw,kf. 191941

5 ((urgent care or emergency) adj2 (room* or service* or department*)).tw,kf. 168894

6 3 or 4 or 5 403147

7 exp "appointments and schedules"/ 23000

8 (wait* time* or efficien* or schedul* or predict*).tw,kf. 3424366

9 (patient flow or work flow or work design or workflow).tw,kf. 394
43

10 (physician initial assessment or PIA or left without being seen or LWBS or length of stay or LOS).tw,kf. 135237

11 7 or 8 or 9 or 10 3575965

12 (natural language process* or computer vision or robotic* or neural network*or generative adversarial network* or image classification or object detection or image processing or case-based reasoning or game playing or transformer* or swarm intelligence or gradient descent or simulated annealing or decision tree* or random forest* or support vector machines or k-nearest neighbor* or k-nearest neighbour* or naive bayes or gradient boosting machines or xgboost or tokenization or policy gradient method* or autoregressive integrated moving average or support vector machine* or Bayesian network* or k-mean* or hierarchical cluster* or association rule mining).tw,kf. 191518

13 ((Data or text) adj1 mining).tw,kf. 18021
14 ((supervised or unsupervised or transfer or semi-supervised or active) adj1 learning).tw,kf. 17382
15 (AI or CNN or RNN or GAN or NER or SGD or GA or PPO or BERT or GPT or ARIMA or SARIMA or SVM or DBSCAN).tw,kf. 159718
16 ((named entity or speech) adj1 recognition).tw,kf. 5877
17 ((expert or rule-based or fuzzy or knowledge-based or decision support) adj1 systems).tw,kf. 6706
18 ((evolutionary or cognitive) adj1 computing).tw,kf. 226
19 ((ant colony or particle swarm or proximal policy) adj1 optimization).tw,kf. 3873
20 ((genetic or simplex or apriori) adj1 algorithm).tw,kf. 9343
21 ((linear or logistic) adj1 regression).tw,kf. 533643
22 1 or 2 or 12 or 13 or 14 or 15 or 16 or 17 or 18 or 19 or 20 or 21 1040557
23 6 and 11 and 22 9895
