# Supplementary material for: Applications of Artificial Intelligence in Emergency Departments to Improve Wait Times: Protocol for an Integrative Living Review
Source: JMIR Res Protoc. 2024 Apr 12;13:e52612. doi: 10.2196/52612 (PMC11053385; doi:10.2196/52612)
Supplement: Multimedia Appendix 2 [file resprot_v13i1e52612_app2.docx]

AI-related concepts and terminology:

- **Machine Learning Algorithms:**
- Supervised Learning:
  - Linear Regression
  - Logistic Regression
  - Support Vector Machines (SVM)
  - Decision Trees
  - Random Forests
  - k-Nearest Neighbors (k-NN)
  - Neural Networks
- Unsupervised Learning:
  - K-Means Clustering
  - Hierarchical Clustering
  - Principal Component Analysis (PCA)
  - Independent Component Analysis (ICA)
  - Autoencoders
- Reinforcement Learning:
  - Q-Learning
  - Deep Q Network (DQN)
  - Policy Gradient Methods
  - Actor-Critic
- **Natural Language Processing (NLP) Algorithms:**
  - Tokenization
  - Named Entity Recognition (NER)
  - Part-of-Speech (POS) tagging
  - Word Embeddings (e.g., Word2Vec, GloVe)
  - Transformer Models (e.g., BERT, GPT)
- **Computer Vision Algorithms:**
  - Haar Cascades
  - Convolutional Neural Networks (CNN)
  - Region-based CNN (R-CNN)
  - YOLO (You Only Look Once)
  - Image Segmentation (e.g., U-Net)
- **Evolutionary Algorithms:**
  - Genetic Algorithms
  - Genetic Programming
  - Differential Evolution
- **Clustering Algorithms:**
  - K-Means
  - DBSCAN (Density-Based Spatial Clustering of Applications with Noise)
  - Agglomerative Hierarchical Clustering
- **Swarm Intelligence Algorithms:**
  - Particle Swarm Optimization (PSO)
  - Ant Colony Optimization (ACO)
- **Optimization Algorithms:**
  - Gradient Descent
  - Stochastic Gradient Descent (SGD)
  - Adam
  - RMSprop
- **Deep Learning Architectures:**
  - Feedforward Neural Networks
  - Recurrent Neural Networks (RNN)
  - Long Short-Term Memory (LSTM)
  - Gated Recurrent Unit (GRU)
  - Attention Mechanism
- **Fuzzy Logic Systems:**
  - Fuzzy Inference System (FIS)
  - Mamdani and Sugeno models
- **Ensemble Learning Algorithms:**
  - Bagging (e.g., Bootstrap Aggregating)
  - Boosting (e.g., AdaBoost, Gradient Boosting)
- **Instance-based Learning:**
  - k-Nearest Neighbors (k-NN)
- **Bayesian Algorithms:**
  - Naive Bayes Classifier
  - Bayesian Networks

**Other AI related terms:**

- Robotics
- Data Mining
- Expert Systems
- Evolutionary Computing
- Knowledge-Based Systems
- Decision Support Systems
- Cognitive Computing
- Transfer Learning
- Semi-Supervised Learning
- Active Learning
- Generative Adversarial Networks (GAN)
- Transformers
- Association Rule
- Time series
- Game AI
- XGBoost
- Time Series Analysis
  - Autoregressive Integrated Moving Average (ARIMA)
  - Seasonal Autoregressive Integrated Moving-Average (SARIMA)
- Apriori Algorithm
- Simulated Annealing
- Speech Recognition
- Sentiment Analysis
- Etc.
