# Supplementary material for: Applications of Artificial Intelligence in Emergency Departments to Improve Wait Times: Protocol for an Integrative Living Review
Source: JMIR Res Protoc. 2024 Apr 12;13:e52612. doi: 10.2196/52612 (PMC11053385; doi:10.2196/52612)
Supplement: Multimedia Appendix 3 [file resprot_v13i1e52612_app3.docx]

ChatGPT 3.5

**Command 1: “What are the subsets of AI?”**

ChatGPT

There are numerous subsets of AI, including:

Machine Learning (ML)

Deep Learning (DL)

Natural Language Processing (NLP)

Computer Vision (CV)

Robotics

Expert Systems

Autonomous Vehicles

Reinforcement Learning

Generative Adversarial Networks (GANs)

Cognitive Computing

Knowledge Representation and Reasoning

Planning and Scheduling

Fuzzy Logic

Evolutionary Computation

Speech Recognition

Emotion AI

AI Ethics and Bias

AI for Healthcare

AI for Finance

AI for Gaming

And many more. These subsets often overlap and intersect with each other, contributing to the multidisciplinary nature of artificial intelligence.

**Command 2: “What are the AI algorithms?”**

ChatGPT

There are various AI algorithms used across different subsets of artificial intelligence. Some common AI algorithms include:

Linear Regression: Used in supervised learning for predicting continuous values.

Logistic Regression: Used in classification tasks to predict binary outcomes.

Decision Trees: Used for classification and regression tasks by partitioning data into subsets based on features.

Random Forest: Ensemble learning method that combines multiple decision trees to improve accuracy and reduce overfitting.

Support Vector Machines (SVM): Used for classification and regression tasks by finding the optimal hyperplane that separates data into different classes.

K-Nearest Neighbors (KNN): A non-parametric method used for classification and regression tasks based on the similarity of data points.

K-Means Clustering: Unsupervised learning algorithm used for clustering data into groups based on similarity.

Neural Networks: Computational models inspired by the structure and function of the human brain, used for various tasks including classification, regression, and pattern recognition.

Convolutional Neural Networks (CNNs): Specialized neural networks commonly used for computer vision tasks due to their ability to capture spatial hierarchies in data.

Recurrent Neural Networks (RNNs): Neural networks designed to work with sequence data, commonly used in natural language processing and time series analysis.

Long Short-Term Memory (LSTM): A type of RNN that is capable of learning long-term dependencies, often used for sequence prediction tasks.

Genetic Algorithms: Optimization algorithms inspired by the process of natural selection, used for optimization and search problems.

Ant Colony Optimization (ACO): Metaheuristic optimization algorithm inspired by the foraging behavior of ants, used for solving combinatorial optimization problems.

Particle Swarm Optimization (PSO): Optimization algorithm inspired by the social behavior of birds flocking or fish schooling, used for optimization problems.

Bayesian Networks: Probabilistic graphical models used for reasoning under uncertainty and making predictions based on probabilistic relationships between variables.
